# Medical and Philosophical News

**Published:** 1781-03

**Authors:** 


At a late monthly meeting of the College of Physicians at Paris, M. le Clerc related a case of phthisis pulmonalis, which had come on in consequence of the sudden repulsion of a cutaneous eruption, and advanced with singular rapidity, so that the patient was in the last stage of consumption, although the cough had begun only about a fortnight before.— M. Bourdois de la Motte read the cases of several unfortunate patients who had fallen victims to an improper use of Keyser's pills, and one of the physicians to the Hotel Dieu took this occasion to remark, that at the time when these pills were the most in vogue, a great number of consumptive patients came into that hospital who owed their complaints to that remedy.

